# Social Environmental Antecedents of Athletes’ Emotions

**DOI:** 10.3390/ijerph18094997

**Published:** 2021-05-08

**Authors:** Montse C. Ruiz, Paul R. Appleton, Joan L. Duda, Laura Bortoli, Claudio Robazza

**Affiliations:** 1 Faculty of Sport and Health Sciences, University of Jyväskylä, 40014 Jyväskylä, Finland; 2 School of Sport, Exercise and Rehabilitation Sciences, College of Life and Environmental Sciences, University of Birmingham, Edgbaston Birmingham, Birmingham B15 2TT, UK; P.Appleton@bham.ac.uk (P.R.A.); J.L.Duda@bham.ac.uk (J.L.D.); 3 BIND-Behavioral Imaging and Neural Dynamics Center, Department of Medicine and Aging Sciences, “G. d’Annunzio” University of Chieti-Pescara, 66013 Chieti, Italy; l.bortoli@unich.it (L.B.); c.robazza@unich.it (C.R.)

**Keywords:** emotions, mediation, motivation, structural equation modeling

## Abstract

The coach-created motivational climate influences variations in athletes’ motivation and emotional experiences. The present study aimed to examine social environmental antecedents of athletes’ emotions. Participants (*N* = 262, 52% female, *M* age = 22.75 ± 6.92) completed questionnaires assessing perceptions of coach-created motivational climates, goal orientations, motivation regulations, and emotions. The mediation effects of goal orientations (i.e., task/ego) and motivation regulations (i.e., autonomous/controlled) on the relationship between motivational climate (i.e., empowering/disempowering) and emotions (i.e., happiness, excitement, anxiety, dejection, and anger) were examined. Structural equation modeling revealed positive direct effects of perceptions of an empowering motivational climate on happiness. Indirect effects of empowering climate to happiness and excitement via task orientation and autonomous motivation emerged. Perceptions of a disempowering climate positively predicted anxiety, dejection, and anger via ego orientation and controlled motivation. Overall, the findings have implications for coach education as they highlight the importance of creating more empowering environments and avoiding or reducing social comparisons.

## 1. Introduction

Athletes’ experiences associated with performance have important repercussions for their performance and wellbeing [1,2]. In sport, one of the social psychological predictors of athletes’ wellbeing and functioning is the coach-created motivational climate, which influences variations in motivation quality as well as emotional experiences [3]. This study aimed to further the understanding of the social environmental factors influencing athletes’ emotional experiences.

The social psychological environment such as the one created by the coach is an important factor shaping athletes’ experiences and their quality of sport engagement [3,4]. According to achievement goal theory [4], perceived motivational climate is a feature of the social environment with different implications depending on whether an individual is more or less task- or ego-involved. Athletes perceive a task-involving climate in environments where coaches emphasize effort, accept errors as part of learning, and focus on improvement and cooperation. In contrast, coaches who emphasize comparison based on ability and rivalry, evaluate progress in terms of outperforming others, and provide differential attention and recognition to athletes of different abilities create an ego-involving climate. In achievement goal theory, two types of dispositional goal orientations are distinguished depending on the individual tendency to emphasize task- or ego-focused criteria to define success. Highly task-oriented individuals focus on improvement and have a self-referenced perception of their competence, whereas ego-oriented individuals focus on outperforming others, reflecting an other-referenced perception of competence. Research has shown that perceptions of a task-involving climate are associated with intrinsic motivation, task orientation, increased effort, and pleasant affective experiences, whereas perceptions of an ego-involving climate are linked with ego orientation, extrinsic motivation, and negative affect [5,6,7,8].

According to self-determination theory [3], the social environment created by the coach, in terms of being more or less autonomy-supportive or controlling, is associated with variations in the quality of motivation. Self-determination theory postulates that individuals’ motivation regulations can be placed on a continuum spanning from most self-determined to lack of motivation. Most self-determined or autonomous forms of motivation refer to engaging in an activity out of pleasure and choice as individuals understand and accept the value of the activity. Less self-determined or controlled motivation, in contrast, derives from engagement in an activity for internal or external pressures indicating that contingencies of self-worth control the individuals. Autonomy-supportive environments, that is, those environments in which athletes’ preferences and feelings are acknowledged, and socially supportive environments, in which athletes feel cared for and valued [9], are supportive of their basic psychological needs of autonomy, competence, and relatedness. In contrast, in controlling coaching environments athletes feel pressured, coerced, or intimidated [10]. More autonomy-supportive and less controlling environments are expected to result in optimal engagement and autonomous motivation.

Considering the different features of the motivational climates (i.e., task- and ego-involving, autonomy-supportive and controlling) together rather than separately is believed to provide a fuller understanding of the influence of the coach-created impact on athletes’ experiences [11]. Drawing from achievement goal theory [4] and self-determination theory [3], Duda et al. [12,13,14] proposed a hierarchical conceptualization of the motivational climate created by the coach that integrates the major social environmental dimensions emphasized within both theories. According to this conceptualization, the motivational climate created by the coach is multidimensional and can be considered to be more or less empowering and disempowering. The empowering feature of the motivational climate includes task-involvement, autonomy-supportive, and socially-supportive characteristics, whereas the disempowering feature of motivational climate is related to perceptions of the social environment as ego-involving and controlling.

Empowering climates are assumed to satisfy individuals’ basic psychological needs, and to be associated with autonomous striving and healthy and sustained engagement in the activity, thus promoting athletes’ quality of engagement and overall health [13]. Conversely, disempowering climates are expected to predict controlled reasons for engagement, and have negative implications for athletes’ experiences and their wellbeing. Research has provided support for these assumptions, showing that facets of an empowering climate are associated with athletes’ autonomous motivation and enjoyment, and negatively related to controlled motivation [15,16]. The multidimensional conceptualization of empowering and disempowering climate extends self-determination theory by distinguishing between competence per se and task-focused competence, suggesting that, in some cases, empowering climates may support basic psychological needs, although this may have some detrimental consequences when competence is conceived in an ego-involving manner [11].

### Study Purpose

Framed within empowering and disempowering motivational climates [12,13,14], the purpose of the study was to examine the interplay between athletes’ perceptions of coach-created empowering and disempowering motivational climates, achievement goal orientations (i.e., task- and ego-orientation), motivation regulations (i.e., autonomous motivation, controlled motivation), and emotions (i.e., happiness, excitement, anxiety, dejection, and anger). We hypothesized that an empowering climate would positively relate to task-orientation, autonomous motivation, and pleasant emotions (i.e., happiness and excitement), whereas a disempowering climate would positively predict ego-orientation, controlled motivation, and unpleasant emotions (i.e., anxiety, dejection, and anger). We also aimed to investigate the mediation effects of goal orientations and motivation regulations in the relationship between motivational climate and athletes’ emotions.

## 2. Materials and Methods

### 2.1. Sample

In this study, we recruited 281 British athletes (142 female, 139 male) from a variety of team (*n* = 165) and individual (*n* = 116) sports. Participants’ age ranged from 17 to 57 years (*M* = 22.76, *SD* = 6.95); one athlete did not specify their age. Ninety-seven participants were national level, and 181 regional level competitors. Three participants did not indicate their competitive level. They had been practicing their sport for an average of 10.30 years (*SD* = 6.41). The mean number of years at the current club/team was 4.44 years (*SD* = 5.06).

### 2.2. Measures

#### 2.2.1. Motivational Climate

Participants’ perceptions of coach-created empowering and disempowering features of the motivational climate were measured on the empowering and disempowering motivational climate questionnaire (EDMCQ-C) [11]. The EDMCQ-C comprises 34 items. Seventeen items measure task-involving (e.g., “My coach tried to make sure players felt good when they tried their best”), autonomy-supportive (e.g., “My coach gave athletes choices and options”) and socially-supportive climates (e.g., “My coach could really be counted on to care, no matter what happened”), and other 17 items measure ego-involving (e.g., “My coach favored some players more than others”) and controlling climates (e.g., “My coach was less accepting of players if they disappointed him or her.”). Participants rated how it has usually been on their team during the last 3–4 weeks on a 5-point Likert scale ranging from 1 (*strongly disagree*) to 5 (*strongly agree*). Adequate internal consistency (e.g., α values >0.86 for each subscale), and convergent and discriminant validity have been reported in samples of younger athletes [11,17].

#### 2.2.2. Motivation Regulations

Motivation regulations were measured using 20 items from the Behavioral Regulation in Sport Questionnaire (BRSQ) [18]. The assessed regulations were intrinsic (e.g., “because I enjoy it”), integrated (e.g., “because it’s a part of who I am”), identified (e.g., “because the benefits of sport are important to me”), introjected (e.g., “because I would feel ashamed if I quit”), and external (e.g., “because people push me to play”), which were measured on four items each. Participants assessed why they participated in their sport on a 7-point Likert scale ranging from 1 (*not at all true*) to 7 (*very true*). Following suggestions by Ryan and Connell [19], in previous research, composite scores have been calculated to form autonomous (using items from intrinsic, integrated, and identified regulations) and controlled (introjected and external regulations) motivation styles. Good reliability values of the BRSQ have been reported in athletes [20], with Cronbach α and composite reliability values >0.86 (autonomous motivation) and 0.88 (controlled motivation), also across a three-month period [21].

#### 2.2.3. Goal Orientations

Participants’ dispositional goal orientations were assessed on the Task and Ego Orientation Questionnaire (TEOSQ) [22], which includes 13 items measuring task (seven items) and ego (six items) orientations. Participants indicated their agreement with task-oriented (“I feel most successful in sport when I learn a new skill by trying hard”) or ego-oriented (“I feel most successful in sport when I can do better than my teammates”) items. Responses are rated on a 5-point Likert scale ranging from 1 (*strongly disagree*) to 5 (*strongly agree*). High internal consistency with Cronbach α values >0.80 (task orientation) and >0.84 (ego orientation) and evidence of good validity for the two-factor structure have been reported in samples of athletes [22,23].

#### 2.2.4. Emotions

The Sport Emotion Questionnaire (SEQ) [24] was used to measure pleasant and unpleasant emotions. The SEQ comprises 22 items to assess happiness (“Cheerful”), excitement (“Enthusiastic”), anxiety (“Nervous”), dejection (“Disappointed”), and anger (“Annoyed”). Participants rated their responses on a 5-point Likert scale (0 = *not at all*; 4 = *extremely*) reflecting how they felt in relation to their next performance. Acceptable levels of internal consistency across all subscales (Cronbach α values >0.77) and support for the five-factor solution were reported [25].

### 2.3. Procedure

The study was conducted following approval from the local institution review board at the second and third authors’ University. The participants were recruited from University sport teams, sport federations, and clubs in England. Team coaches and managers were contacted and explained the general purpose of the study to gain access to the participants. Participants were informed of the purpose of the study, that there were no right or wrong answers, and that they could withdraw at any time without any penalty. Confidentiality of individual results and voluntary nature of participation were emphasized. The measures were administered prior to a training session by the first author and research assistants who received relevant preparation on data collection procedures. Questionnaire administration was carried out individually or in small groups, in a quiet place nearby training facilities. Data collection took place a few weeks after the season started to ensure that participants had experience and awareness of relevant aspects of the coach-created environment. The assessment took approximately 30 min.

### 2.4. Data Analysis

Data screening included evaluation of missing data, identification of potential univariate and multivariate outliers, and examination of assumptions of normality, homoscedasticity, and linearity. Descriptive statistics, Pearson product-moment correlation coefficients, and reliability via Cronbach α, omega (ω) [26], and composite reliability (CR) values were calculated for all the study variables (i.e., empowering climate, disempowering climate, autonomous motivation, controlled motivation, task orientation, ego orientation, happiness, excitement, anxiety, dejection, and anger). Values greater than 0.70 are indicative of adequate reliability. Correlation coefficients were interpreted following Zhu’s [27] recommendations, that is, 0–0.19 = no correlation, 0.20–0.39 = low correlation, 0.40–0.59 = moderate correlation, and 0.60–0.79 = moderately high correlation. Average variance extracted (AVE) for the latent variables was computed, with values close to or larger than 0.50 indicative of adequate item convergence [28]. Differences in mean scores in the study variables across gender and competitive level (regional vs. national) were ascertained through multivariate analysis of variance (MANOVA).

Confirmatory factor analyses (CFA) were performed to examine the factorial validity of the measures. CFAs were performed with Mplus 8.5 [29], using the missing-data function and maximum likelihood estimator (MLR) to adjust for non-normality with the robust full information. Model fit was assessed using chi-square (χ^2^), comparative fit index (CFI), Tucker–Lewis fit index (TLI), root mean square error of approximation (RMSEA), and standardized root mean square residual (SRMR). The following indices are representative of a good model fit: CFI and TLI close to 0.95, RMSEA smaller than 0.06, and SRMR smaller than 0.08 [30,31].

Structural equation modeling was performed to test the hypothesized relationships between social environmental antecedents of athletes’ emotions. Specifically, a model was estimated to test the expected relationships between motivational climate and individual motivational processes with athletes’ emotions. The bias-corrected bootstrap method based on 5000 resamples was used to test hypothesized mediator effects. Significant mediation is assumed when zero is not included in the 95% confidence intervals [32].

## 3. Results

Data screening revealed 16 cases with several missing values (>5%). Three cases were identified as outliers based on Mahalanobis’ distance criterion. These cases were removed from further analyses, resulting in a sample of 262 participants (52% female, *M* age = 22.75 ± 6.92, 66% involved in regional level competitions).

Descriptive statistics, Pearson product-moment correlations, reliability indices, and average variance extracted values are presented in Table 1. Overall, acceptable reliability indices, composite reliability, and variance extracted were found. Participants reported higher scores for empowering climate, autonomous motivation, task orientation, and excitement.

MANOVA yielded significant results by competitive level, Pillai’s trace = 0.105, *F*(11, 243) = 2.605, *p* = 0.004, *η*_p_^2^ = 0.105, and by sport, Pillai’s trace = 0.090, *F*(11, 243) = 2.192, *p* = 0.015, *η*_p_^2^ = 0.090. The competitive level by sport interaction was also significant, Pillai’s trace = 0.083, *F*(11, 243) = 1.992, *p* = 0.030, *η*_p_^2^ = 0.083. Univariate follow-up showed that athletes involved in regional competitive level reported significantly higher mean scores on empowering climate, and lower scores in dejection and anger compared with athletes involved in higher competitive level. Moreover, team sport athletes reported significantly lower scores in ego orientation and higher scores in excitement and happiness compared with athletes involved in individual sports. No significant results were observed across gender (*p* = 0.343) or for any of the remaining factor interactions (*p* > 0.392).

CFA analyses conducted using individual items resulted in poor fit to the data for the motivational climate, goal orientations, and motivation regulations measures. Therefore, individual items were combined into construct-specific parcels based on the theoretical structure of each measure. Item parceling has been recommended to increase model parsimony and to improve the ratio of variable to sample size [33,34]. Specifically, in the case of motivational climate, three parcels were formed by calculating the sums of items representing task-involving, autonomy-supportive, and socially-supportive features of motivational climate, distributed equally across parcels forming the empowering climate factor, while the remaining ego-involving and controlling items were assigned to three parcels representing disempowering climate. For goal orientations, three parcels were created using items representing task orientation, and three parcels were created with the remaining items representing ego orientation. Regarding motivation regulations, four parcels included items representing intrinsic motivation, integrated regulation, and identified regulation scales, distributed equally across parcels as indicators of autonomous motivation, and four parcels were calculated from introjected regulation and external regulation subscales as indicators of controlled motivation. CFA analysis for the emotions measure yielded good fit to the data. Factor loadings for all measures were >0.40. CFA results are reported in Table 2. 

**Figure 1:**
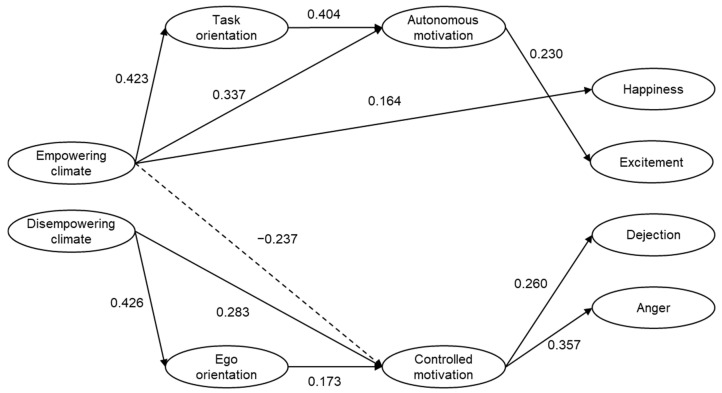
Structural equation model illustrating interrelationships between perceived motivational climate, goal orientations, motivation regulations, and emotions. Significant standardized estimates are presented (*p* < 0.05). Anxiety is not depicted as no significant direct paths emerged.

SEM analyses were conducted to examine the relationships between motivational climate, goals orientations, motivation regulations, and emotions. We controlled for competitive level (national, regional) and for sport modality (team, individual) by entering them as covariates. After examination of modification indices, the path from empowering climate to controlled motivation was added. The model fitted the data well, χ^2^(862) = 1399.452, CFI = 0.917, TLI = 0.909, RMSEA = 0.049 (90% CI = 0.044−0.053), SRMR = 0.073. Significant path coefficients are presented in Figure 1. As expected, empowering climate was found to be a positive predictor of task orientation, autonomous motivation, and happiness. A significant negative path from empowering climate to controlled motivation emerged, which is also in line with theoretical expectations. Disempowering climate was found to be a positive predictor of ego orientation and controlled motivation, which in turn positively predicted dejection and anger. The direct paths to anxiety were not significant.

Mediation analysis demonstrated that perceptions of an empowering climate had a significant positive indirect effect on happiness, via autonomous motivation. Significant positive effects of perceptions of empowering climate on excitement, via autonomous motivation alone, and via task orientation and autonomous motivation, were observed. A significant positive indirect effect emerged from perceptions of a disempowering climate on anxiety, via controlled motivation. A significant positive indirect effect from perceptions of a disempowering climate to anxiety via ego orientation and controlled motivation was also observed. A positive indirect effect emerged for perceptions of a disempowering climate on dejection, via controlled motivation. A significant positive indirect effect also emerged from perceptions of a disempowering climate on dejection, via ego orientation and controlled motivation. Similarly, results indicated positive indirect effects of perceptions of a disempowering climate on anger, via controlled motivation alone, and via ego orientation and controlled motivation (see Table 3).

## 4. Discussion

Pulling from achievement goal theory [4], self-determination theory [3], and the integrated conceptualization of the motivational climate [12,13,14], this study investigated the social environmental antecedents of emotional experiences in male and female athletes. In particular, we examined the relationships between perceptions of empowering and disempowering features of coach-created motivational climates, goal orientations, motivation regulations, and athletes’ pleasant (i.e., happiness, excitement) and unpleasant emotions (i.e., anxiety, dejection, anger). The hypothesized mediating effects of goal orientations and motivation regulations were tested. Overall, our findings are consistent with the theoretical assumptions [3,4,12] and extend the previous literature on the interplay between the coach-created motivational climate and variations in athletes’ goal orientations, motivation quality, and emotional experiences.

Athletes reported higher values for empowering motivational climate, task orientation, autonomous motivation, and pleasant emotions, whereas the reported values were lower for disempowering climate, ego orientation, controlled motivation, and the unpleasant emotions (Table 2). A moderate negative correlation emerged between both features of motivational climate.

### 4.1. Empowering Motivational Climate

Our findings showed that perceptions of an empowering motivational climate (task-involving, autonomy and socially supportive) were associated with adaptive motivational and emotional outcomes for the athletes. Specifically, structural equation modeling revealed significant direct positive effects between perceptions of an empowering motivational climate on happiness (Figure 1). Contrary to our hypothesis, the path from empowering motivational climate to excitement was not significant. Although happiness and excitement are both positively toned emotions, the results may reflect the different intensity level of their manifestation. Happiness is akin to joy but considered to be a low intensity emotion, while excitement is typically regarded as a high intensity emotion [24]. Overall results suggest that perceptions of a motivational climate as empowering can foster positive experiences in athletes.

Support for the hypothesized links between empowering climate with task orientation and autonomous motivation was found. A negative association between empowering climate and controlled motivation was also observed. These results are in line with the self-determination theory tenets [3] and Duda et al.’s [12,13,14] conceptualization of the motivational climate created by the coach, which posits that climates that emphasize task-orientation, autonomy, and social support lead to more positive outcomes.

### 4.2. Disempowering Motivational Climate

In line with our hypothesis, perceptions of a disempowering motivational climate were positive predictors of ego goal orientations and controlled motivations. However, the direct paths from disempowering climate to the three unpleasant emotions assessed were not significant. Controlled motivation was a positive predictor of dejection and anger. No significant paths emerged to anxiety. In seeking to explain the lack of significant paths to anxiety, we can argue that this could be due to the different ways participants may interpret anxiety. For instance, anxiety can be perceived as facilitative or debilitative for performance depending on how athletes appraise the situation in terms of potential anticipated gains or loses. This notion has been supported by a large body of empirical evidence [2,35,36].

### 4.3. Mediation Analysis

Our results demonstrate that empowering climate is positively related to happiness and excitement, via a positive association with autonomous motivation (Table 3). A sequential positive association emerged between empowering climate and excitement, via task orientation and autonomous motivation. The existence of positive indirect effects of empowering climate on excitement via task orientation and autonomous motivation is in line with the notion that excitement reflects athletes’ positive expectations of their ability to cope and attain their goals [24].

These results underline the adaptive features of promoting environments where coaches provide meaningful choices and recognize athletes’ feelings, and where athletes feel valued. Emphasizing autonomous motives for participation and focusing on improvement rather than social comparison are important for the experience of pleasant emotions amongst male and female athletes. This contention has been well-substantiated in the literature, with research showing that athletes’ perceptions of an empowering motivational climate lead to more self-determined forms of motivation and pleasant emotional experiences [15,37,38,39].

Our findings suggest that disempowering climate is positively associated with anxiety, dejection, and anger, via a positive association with controlled motivation. A sequential positive association between disempowering climate and all the unpleasant emotions, ego orientation, and controlled motivation was also found. The findings support the notion of detrimental effects of disempowering climate which can result in athletes’ feelings of deficiency and sadness, anxiety, and anger, which could be channeled inwardly (e.g., self-blame) or outwardly and result in aggressive behavior. The findings are in line with theoretical propositions [3,4,12] and evidence on the positive associations of ego-involving and controlling coaching climates with burnout, disordered eating, depression, anxiety, and anger [20,21,40,41]. These results highlight the negative implications of a focus on external reward or punishment contingencies and social comparison, which are associated with unpleasant emotions in male and female athletes.

### 4.4. Practical Implications

Taken together, our results suggest that the adaptive effects of empowering climate were more prevalent, with direct effects on happiness, whereas ego orientation and engagement in the activity for controlled motives mediated the detrimental effects of disempowering climate, which may lead to athletes’ less-than-optimal functioning and ill-being.

Our findings highlight the importance of promoting environments where coaches provide athletes with opportunities to take the initiative, focus on their development, and acknowledge their feelings, while minimizing social comparison and avoiding controlling statements and behaviors. These findings emphasize the importance of providing coaches with training aimed at helping them reduce social comparisons and avoid controlling coaching styles, as well as promoting more adaptive environments in which coaches provide meaningful choices, consider their athletes’ input, and recognize their effort and progress [16]. An example of an evidence-based program is Empowering Coaching™ [14].

For coaches to acknowledge athletes’ emotions, they should accurately perceive and be able to identify their athletes’ experiences [42]. How coaches perceive their athletes’ emotional experiences and use such information has important implications in the development of environments conducive to athletes’ wellbeing and sustainable performance. This area of research, however, has received limited attention. Exploratory studies suggest that coaches who are accurate in identifying their athletes’ emotional experiences, managing their own emotions accordingly, are perceived as caring and interested, which may be helpful for their own psychological states and performance [43,44]. Several strategies can be used to help coaches develop and enhance their emotional competences [45].

### 4.5. Study Limitations and Future Research Directions

While this study has strengths, we have to acknowledge its limitations. The cross-sectional nature of the study cannot provide insight into the temporal ordering of the studied variables. Based on findings from previous research [21] with task- and ego-involving motivational climates, it can be assumed that the empowering and disempowering climates also have carryover effects on athletes’ quality of motivation and emotional experiences. However, future research including longitudinal designs or experimental studies are warranted to further understand the interplay among social environmental factors and athletes’ motivation and emotional experiences. The use of item parceling might be viewed as another limitation. However, we deemed item parceling appropriate in this study to improve the variable to sample size ratio. Indeed, this statistical strategy has been recommended with small sample size studies to increase model parsimony and parameter estimates stability [34]. Future research using individual items as indicators of the underlying constructs in studies including larger samples of athletes of different competitive levels and sport disciplines is warranted. Another limitation of this study is that we assessed a limited number of emotions. While we included both pleasant and unpleasant emotions, athletes go through different experiences associated with performance which we did not capture. Further research could aim at examining the relationships between coach-created social environments and a wide range of performance-related experiences.

## 5. Conclusions

In summary, perceptions of an empowering motivational climate were positive predictors of task orientation, self-determined forms of motivation, and happiness. Conversely, perceptions of a motivational climate as disempowering were positive predictors of ego goal orientation and/or controlled motivation. No direct links were observed from disempowering coaching and unpleasant emotions. Task orientation and autonomous motivation mediated the effects of empowering coaching on happiness and excitement. Ego orientation and/or controlled motivation mediated the effects of disempowering climate on athletes’ unpleasant emotions (i.e., anxiety, dejection, and anger).

The results from this study indicate that the type of motivational climate created by the coach has important consequences in terms of athletes’ motivation as well as their emotional experiences. Our results highlight the importance of the coach, and the creation of an adaptive motivational climate that would reduce social comparisons and avoid controlling coaching styles. In addition, the study extends the literature on the antecedents of athletes’ emotions.

## Figures and Tables

**Table 1:** Descriptive statistics, Pearson product-moment correlation coefficients, and reliability indices (*N* = 262).

Variable	*M* ± *SD*	1	2	3	4	5	6	7	8	9	10	α	CR	ω	AVE
1. Empowering climate	3.86 ± 0.56											0.906	0.917	0.907	0.984
2. Disempowering climate	2.55 ± 0.63	−0.402 ^§^										0.869	0.901	0.872	0.976
3. Task orientation	4.07 ± 0.55	0.420 ^§^	−0.193									0.820	0.843	0.825	0.938
4. Ego orientation	2.79 ± 0.91	−0.204 *	0.364 *	0.030								0.859	0.845	0.861	0.932
5. Autonomous motivation	4.16 ± 0.52	0.426 ^§^	−0.230 *	0.537 ^§^	−0.066							0.860	0.842	0.814	0.877
6. Controlled motivation	2.09 ± 0.83	−0.396 *	0.448 ^§^	−0.297 *	0.274 *	−0.348 *						0.926	0.906	0.920	0.962
7. Happiness	2.27 ± 0.86	0.219 *	−0.041	0.178 *	−0.153	0.246 *	−0.199					0.854	0.852	0.855	0.915
8. Excitement	2.39 ± 0.79	0.187	−0.025	0.203 *	−0.063	0.245 *	−0.200 *	0.594 ^§^				0.776	0.788	0.786	0.811
9. Anxiety	1.37 ± 0.99	0.035	0.081	0.101	0.113	−0.025	0.152	−0.166	0.200 *			0.899	0.899	0.905	0.936
10. Dejection	0.32 ± 0.58	−0.184	0.190	−0.142	0.119	−0.206 *	0.374 *	−0.167	−0.255 *	0.262 *		0.858	0.863	0.857	0.678
11. Anger	0.40 ± 0.64	−0.220 *	0.158	−0.177	0.130	−0.209 *	0.396 *	−0.096	−0.191	0.206 *	0.796 ^†^	0.846	0.853	0.847	0.914

Note. α = Cronbach’s alpha values, ω = omega values, CR = composite reliability, AVE = average variance extracted; * low correlation, ^§^ moderate correlation, ^†^ moderately high correlation.

**Table 2:** Fit indices for each studied variable derived from confirmatory factor analyses.

Measures	*χ*^2^(df)	CFI	TLI	RMSEA (90% CI)	SRMR
Coach-created motivational climate (34 items, 2 factors)	801.563 (463)	0.869	0.860	0.053 (0.047–0.059)	0.068
Coach-created motivational climate (34 items, 6 parcels)	11.039 (8)	0.997	0.994	0.038 (0.000–0.088)	0.023
Goal orientation (13 items, 2 factors)	206.207 (64)	0.863	0.832	0.092 (0.078–0.106)	0.066
Goal orientation (13 items, 6 parcels)	11.650 (8)	0.994	0.988	0.042 (0.000–0.090)	0.034
Motivation regulations (20 items, 2 factors)	396.062 (160)	0.878	0.856	0.075 (0.066–0.084)	0.071
Motivation regulations (20 items, 8 parcels)	32.140 (18)	0.985	0.976	0.055 (0.021–0.085)	0.034
Emotions (22 items, 5 factors)	375.176 (199)	0.924	0.912	0.058 (0.049–0.065)	0.058

Note: *χ*^2^(df) = chi-square (degrees of freedom); CFI = comparative fit index; TLI = Tucker–Lewis fit index; RMSEA = root mean square error of approximation; SRMR = standardized root mean square residual.

**Table 3:** Total, direct, and indirect effects for paths from empowering and disempowering climates to emotions via task- or ego-goal orientations, and autonomous or controlled motivations.

Effect	*β*	*SE*	Bootstrap Bias-Corrected 95% CI (Lower, Upper)	
Empowering climate to Happiness				
Total	0.280 *	0.061	−0.153	0.393
Total indirect	0.115 *	0.047	−0.021	0.207
EC → Task → Happiness	0.018 *	0.042	−0.060	0.099
EC → AM → Happiness	0.065 *	0.041	−0.003	0.168
EC → Task → AM → Happiness	0.033 *	0.022	−0.003	0.086
EC → Happiness	0.164 *	0.074	−0.022	0.310
Empowering climate to Excitement				
Total	0.264 *	0.066	−0.126	0.388
Total indirect	0.132 *	0.049	−0.004	0.233
EC → Task → Excitement	0.015 *	0.046	−0.084	0.099
EC → AM → Excitement	0.077 *	0.038	−0.019	0.174
EC → Task → AM → Excitement	0.039 *	0.022	−0.007	0.099
EC → Excitement	0.132 *	0.077	−0.014	0.289
Disempowering climate to Anxiety				
Total	0.032 *	0.077	−0.112	0.187
Total indirect	0.084 *	0.043	−0.006	0.173
DC → Ego → Anxiety	0.033 *	0.035	−0.033	0.108
DC → CM → Anxiety	0.040 *	0.026	−0.001	0.108
DC → Ego → CM → Anxiety	0.011 *	0.008	−0.001	0.036
DC → Anxiety	−0.052 **	0.091	−0.223	0.139
Disempowering climate to Dejection				
Total	0.229 *	0.071	−0.092	0.365
Total indirect	0.097 *	0.045	−0.010	0.190
DC → Ego → Dejection	0.004 *	0.041	−0.081	0.080
DC → CM → Dejection	0.074 *	0.034	−0.021	0.161
DC → Ego → CM → Dejection	0.019 *	0.011	−0.004	0.050
DC → Dejection	0.132 *	0.083	−0.027	0.292
Disempowering climate to Anger				
Total	0.196 *	0.068	−0.064	0.332
Total indirect	0.159 *	0.047	−0.070	0.265
DC → Ego → Anger	0.031 *	0.036	−0.035	0.107
DC → CM → Anger	0.101 *	0.037	−0.044	0.191
DC → Ego → CM → Anger	0.025 *	0.013	−0.006	0.061
DC → Anger	0.037 *	0.081	−0.127	0.193

Note: * Significance indicated via 95% CI. Abbreviations: *β* = standardized estimate; *SE* = Standard error; CI = Confidence interval; EC = Empowering climate; DC = Disempowering climate; AM = Autonomous motivation; CM = Controlled motivation.

## Data Availability

The data presented in this study are available on request from the corresponding author.
