# Correction: Ruiz et al. Social Environmental Antecedents of Athletes’ Emotions. *Int. J. Environ. Res. Public Health* 2021, *18*, 4997

**DOI:** 10.3390/ijerph18136756

**Published:** 2021-06-23

**Authors:** Montse C. Ruiz, Paul R. Appleton, Joan L. Duda, Laura Bortoli, Claudio Robazza

**Affiliations:** 1 Faculty of Sport and Health Sciences, University of Jyväskylä, 40014 Jyväskylä, Finland; 2 School of Sport, Exercise and Rehabilitation Sciences, College of Life and Environmental Sciences, University of Birmingham, Edgbaston Birmingham, Birmingham B15 2TT, UK; P.Appleton@bham.ac.uk (P.R.A.); J.L.Duda@bham.ac.uk (J.L.D.); 3 BIND-Behavioral Imaging and Neural Dynamics Center, Department of Medicine and Aging Sciences, “G. d’Annunzio” University of Chieti-Pescara, 66013 Chieti, Italy; l.bortoli@unich.it (L.B.); c.robazza@unich.it (C.R.)

In the original article [1], Table 3 did not display properly. The corrected version of Table 3 appears below: 

We would like to apologize for any inconvenience caused to the readers by these changes. The error appeared in the article from 8 May 2021 to 23 June 2021. The original article has been updated.

## Figures and Tables

**Table 3:** Total, direct, and indirect effects for paths from empowering and disempowering climates to emotions via task- or ego-goal orientations, and autonomous or controlled motivations.

Effect	*β*	*SE*	Bootstrap Bias-Corrected 95% CI (Lower Upper)	
Empowering climate to Happiness				
Total	0.280 *	0.061	0.153	0.393
Total indirect	0.115 *	0.047	0.021	0.207
EC → Task → Happiness	0.018	0.042	−0.060	0.099
EC → AM → Happiness	0.065 *	0.041	0.003	0.168
EC → Task → AM → Happiness	0.033	0.022	−0.003	0.086
EC → Happiness	0.164 *	0.074	0.022	0.310
Empowering climate to Excitement				
Total	0.264 *	0.066	0.126	0.388
Total indirect	0.132 *	0.049	0.004	0.233
EC → Task → Excitement	0.015	0.046	−0.084	0.099
EC → AM → Excitement	0.077 *	0.038	0.019	0.174
EC → Task → AM → Excitement	0.039 *	0.022	0.007	0.099
EC → Excitement	0.132	0.077	−0.014	0.289
Disempowering climate to Anxiety				
Total	0.032	0.077	−0.112	0.187
Total indirect	0.084 *	0.043	0.006	0.173
DC → Ego → Anxiety	0.033	0.035	−0.033	0.108
DC → CM → Anxiety	0.040 *	0.026	0.001	0.108
DC → Ego → CM → Anxiety	0.011 *	0.008	0.001	0.036
DC → Anxiety	−0.052	0.091	−0.223	0.139
Disempowering climate to Dejection				
Total	0.229 *	0.071	0.092	0.365
Total indirect	0.097 *	0.045	0.010	0.190
DC → Ego → Dejection	0.004	0.041	−0.081	0.080
DC → CM → Dejection	0.074 *	0.034	0.021	0.161
DC → Ego → CM → Dejection	0.019 *	0.011	0.004	0.050
DC → Dejection	0.132	0.083	−0.027	0.292
Disempowering climate to Anger				
Total	0.196 *	0.068	0.064	0.332
Total indirect	0.159 *	0.047	0.070	0.265
DC → Ego → Anger	0.031	0.036	−0.035	0.107
DC → CM → Anger	0.101 *	0.037	0.044	0.191
DC → Ego → CM → Anger	0.025 *	0.013	0.006	0.061
DC → Anger	0.037	0.081	−0.127	0.193

Note. * Significance indicated via 95% CI. Abbreviations: *β* = standardized estimate; *SE* = Standard error; CI = Confidence interval; EC = Empowering climate; DC = Disempowering climate; AM = Autonomous motivation; CM = Controlled motivation.
